# Using Spatial Pattern Analysis to Explore the Relationship between Vulnerability and Resilience to Natural Hazards

**DOI:** 10.3390/ijerph18115634

**Published:** 2021-05-25

**Authors:** Chien-Hao Sung, Shyue-Cherng Liaw

**Affiliations:** Department of Geography, National Taiwan Normal University, Taipei 10610, Taiwan; koehler@ntnu.edu.tw

**Keywords:** vulnerability, resilience, spatially explicit resilience-vulnerability model (SERV), spatial autocorrelation analysis, geographically weighted regression (GWR), spatial difference

## Abstract

This research aims to explore the spatial pattern of vulnerability and resilience to natural hazards in northeastern Taiwan. We apply the spatially explicit resilience-vulnerability model (SERV) to quantify the vulnerability and resilience to natural hazards, including flood and debris flow events, which are the most common natural hazards in our case study area due to the topography and precipitation features. In order to provide a concise result, we apply the principal component analysis (PCA) to aggregate the correlated variables. Moreover, we use the spatial autocorrelation analysis to analyze the spatial pattern and spatial difference. We also adopt the geographically weighted regression (GWR) to validate the effectiveness of SERV. The result of GWR shows that SERV is valid and unbiased. Moreover, the result of spatial autocorrelation analysis shows that the mountain areas are extremely vulnerable and lack enough resilience. In contrast, the urban regions in plain areas show low vulnerability and high resilience. The spatial difference between the mountain and plain areas is significant. The topography is the most significant factor for the spatial difference. The high elevation and steep slopes in mountain areas are significant obstacles for socioeconomic development. This situation causes consequences of high vulnerability and low resilience. The other regions, the urban regions in the plain areas, have favorable topography for socioeconomic development. Eventually, it forms a scenario of low vulnerability and high resilience.

## 1. Introduction

The concept of vulnerability and resilience to natural hazards has been applied in the field of disaster management. Multiple models and indicators have been developed and applied to investigate the vulnerability and resilience to natural hazards [1,2]. However, most of the models and indicators neglect the spatial difference and spatial pattern. Therefore, the result of most of the studies cannot offer a spatial viewpoint. Spatial differences and spatial patterns are unneglectable factors because human is one of the important subjects of disaster events. Characteristics of humans, such as age, wealth, and occupation, are highly variable spatially. Consequently, humans create a spatial difference to affect the vulnerability and resilience to natural hazards [3,4]. The spatial difference significantly influences the vulnerability and resilience to natural hazards. Moreover, environmental factors, such as topography, have a significant influence on humans and spatial differences [4].

The vulnerability to natural hazards means the potential for loss when facing natural hazards [5,6]. It is created by exposure to stresses associated with environmental and social fabrics [6,7]. Environmental fabrics, such as the elevation and slope, create biophysical vulnerability. Social fabrics, such as experience and socioeconomic conditions, generate social vulnerability. Recently study shows that the essences of vulnerability are far more complex and cannot be limited to the identification of deficiencies [8]. Although it should not be limited to a certain identification, there still exists some consensus of vulnerability. In general, it includes four key factors, which are exposure, susceptibility, lack of resilience or response, and hazard [8,9,10,11,12,13,14]. Moreover, vulnerability is also involved in six dimensions, which are social, economic, physical, cultural, environmental, and institutional [8,9,10,11,12,13,14]. In our research, we integrate the social and economic dimensions and regard it as the social aspect of vulnerability [6,7]. The concept of social vulnerability is highly variable and changing from place to place and over time since it is an interdisciplinary topic with the characteristic of multidimensional and highly dynamic [15,16,17]. In short, social vulnerability topically refers to certain pre-existing conditions of some people, groups and, and organizations when facing environmental stress [18,19,20,21]. These pre-existing conditions are a kind of incapability, which will reduce the preparedness and jeopardize them when facing the impact brought by environmental disturbance [21,22,23]. As for the resilience to natural hazards, the meaning is also still under discussion. Studies show that even resilience to natural hazards still under debate; it contains two major characteristics, which are coping and adaptation [24]. These two characteristics have very different essences. The coping focuses on the current situation of the system and institution while concentrating on constantly learning for a longer period [25,26]. Take the relationship between resilience, vulnerability, and adaptive capacity as an example. Different scholars have different opinions on the linkages between resilience, vulnerability, and adaptive capacity [27,28]. For example, some of the researchers regard vulnerability and resilience as different but link components [29]. Simultaneously, another scholar views resilience as a part of vulnerability [30]. Moreover, some studies define the vulnerability and the adaptive capacity as separate but link components within the resilience [31].

Generally speaking, we can regard resilience to natural hazards as a “bouncing-forward” trajectory [9]. This trajectory not only focuses on recovery but also learning through the experience of natural hazard events [32,33]. Under the circumstance of climate change, merely recovery is not enough because the intensity and frequency of natural hazards will continuously increase. In other words, if we take the resilience to natural hazards as a “bouncing-back” trajectory, it will lead the situation into a devastating outcome in the long run [34,35]. Consequently, in our research, we address the “bouncing-forward” concept as the core concept.

Our research defines adaptive capacity as an element of vulnerability and resilience to natural hazards simultaneously. Because according to the disaster resilience of place (DROP) model, the resilience to natural hazards also includes the concept of adaptive capacity [29]. The system will attempt to adapt and absorb the impact with adaptive capacity when it encounters natural hazards [29]. According to another model, the hazards-of-place model, the vulnerability to natural hazards comprises adaptive capacity. The resilience to natural hazards also contains the positive elements that will decrease social vulnerability [5,36,37,38]. Generally speaking, both vulnerability and resilience to natural hazards are significantly related to adaptive capacity.

The approach to inspecting the vulnerability and resilience to natural hazards can be separated into several types according to the method, spatial scale, and aim [39]. We aim to explore the spatial pattern and difference between vulnerability and resilience to natural hazards at the community scale. As a result, a top-down and quantified approach will be ideal for this research. The spatially explicit resilience-vulnerability (SERV) model is highly modifiable and flexible [40]. The SERV model allows us to quantify vulnerability and resilience for further spatial analysis. We will also use the principal component analysis (PCA) to extract the important factors. Then, we will apply the spatial autocorrelation analysis, which is one of the most idealist statistical approaches, to detect the spatial distribution pattern. Several studies had applied this approach [31,41]. Through the distribution pattern of vulnerability and resilience to natural hazards, we can analyze the spatial difference. For example, under different topography and socioeconomic condition, the vulnerability and resilience to natural hazards could change spatially. In addition, the spatial relationship between vulnerability and resilience to natural hazards could also be different. It is essential to validate whether the result of the SERV model can reflect the ground truth. Therefore, we apply the geographically weighted regression (GWR) to verify the SERV according to the previous studies [31,42].

## 2. Materials and Methods

### 2.1. Yilan County

**Figure 1:**
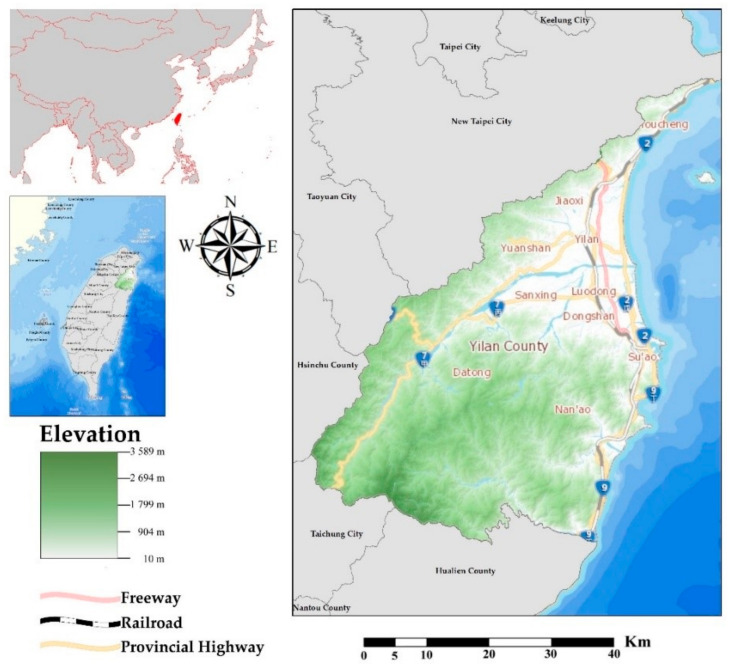
Case study area.

Our case study area is located in northeastern Taiwan, which comprises mountain and plain areas (Figure 1). The elevation of the mountain areas rises up to 3589 m with a very steep slope. Simultaneously, the plain areas are the largest in eastern Taiwan. Yilan County is also the most prosperous county in eastern Taiwan due to the close connectivity with Taipei City. Moreover, the plain areas in Yilan County can be separated into urban and rural regions. The urban regions lay in the center of the plain areas. The industrial structure of the urban regions is mainly the tertiary industrial sector. The industrial structures of the rural and the mountain areas are mostly the primary industrial sector. Yilan County is also extremely vulnerable to natural hazards. Because of the location and distribution of mountain and plain areas, Yilan County is wide-open to typhoons and tropical cyclones coming from the Pacific Ocean. The number of typhoons and tropical cyclones that strike Taiwan is 3 to 4 annually. Over 30% of them will directly influence Yilan County. Furthermore, climate change enhances the intensity and frequency of typhoons and tropical cyclones. This situation increases the amount of precipitation dramatically and the probability of flood and debris flow events in Yilan County.

### 2.2. SERV, Variables Selection, and PCA

The SERV model is an integrated model, which provides us a framework to incorporate vulnerability and resilience together. It composes three main components, which are exposure, sensitivity, and adaptive capacity [3]. Exposure can be regarded as a certain unit that falls within the physical scope of hazard events, which affects not only human systems and social systems but also specific resources and practices [24]. That is, exposure involves spatial and temporal patterns eventually [24]. Sensitivity stands for social vulnerability. Adaptive capacity represents part of resilience. This model focuses on the scale, especially the sub-county level. The following equation demonstrates the relationship between the three main components.

SERV = [E + S] − AC(1)

E: Exposure, S: Sensitivity, AC: Adaptive Capacity.

The variables of SERV can be divided into socioeconomic data and the potential of natural hazards. We obtained the socioeconomic data from the National Geographic Information System (NGIS) Socioeconomic Database. The potential of natural hazards was inquired from the National Science and Technology Center for Disaster Reduction (NCDR) and the Soil and Water Conservation Bureau (SWCB). According to the hazards-of-place model, the vulnerability to natural hazards is the combination of physical (exposure) and social (sensitivity) vulnerability [6]. Consequently, we can regard the [E + S] part as the vulnerability to natural hazards.

We standardized all the variables by the following equation because of the different measurements of all data. This approach can alter the various measurements into the same scale and allows us for further analysis. After standardizing the variables, we classified the values into five categories, which are very high (>1.5 SD), high (1.5 SD–0.5 SD), medium (0.5 SD–−0.5 SD), low (−0.5 SD–−1.5 SD), and very low (<−1.5 SD).

(2) $$\mathrm{Standardization}=\left(X-\bar{X}\right)/\sigma_{X}$$

X: The value of the variable, $\bar{X}$: The mean value of the variables, $\sigma_{X}$: The standard deviation of the variable.

**Figure 2:**
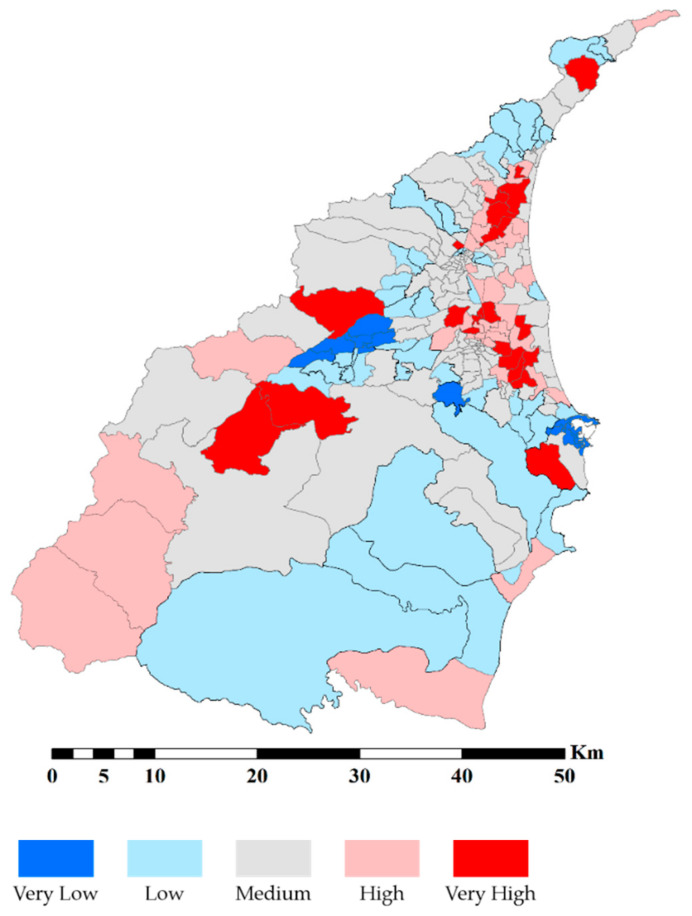
The spatial distribution of exposure to natural hazards.

The most common natural hazards in our case study area are flood and debris flow. In order to properly reflect the authentic exposure, we applied the potential of these two natural hazards inquired from the NCDR and SWCB databases. After the standardization, we combined the potential of these two natural hazards together. Figure 2 demonstrates the spatial distribution of the potential of flood and debris flow hazards.

Subsequently, we selected 12 socioeconomic variables of sensitivity according to the previous research. Table 1 illustrates the socioeconomic variables we adopted. Population density is one of the most cited variables. The population density can depict potential exposure to natural hazards under a certain area [38]. Consequently, scholars consider population density the most effective and general empirical indicators for evaluating social vulnerability [6,43,44]. Studies show the population density is highly correlated to social vulnerability and might be one essential factor for social vulnerability since the higher population density represents a higher requirement during the environmental disturbance [45,46,47]. Some previous research also adopts sex as an important variable [48,49]. Research shows the standardized female population is a significant variable for exploring social vulnerability due to sex inequalities, social responsibility, and limited access to resources [50]. Financial ability is highly related to the ability to cope with natural hazards and evacuation [51]. Generally, insufficient financial ability equal to the inadequate ability to cope with natural hazards [51,52,53]. Accordingly, the middle/low-income (MLI) household is also an important factor [40,52,53]. Moreover, the ratio of dependency has been regarded as a crucial variable. The dependency ratio represents the proportion of the incapacitated population who depend on others [37]. In other words, the dependency ratio means the ratio of population that lacks the ability to facing natural hazards [54]. In addition, the language and culture barriers are also crucial when encountering natural hazards since these abilities exceptionally important for information inquire [40,55]. Consequently, ethnicity is also a significant variable [56]. Therefore, we adopted the indigenous population ratio and foreign residents and laborers to stand for the variables of and ethnicity. According to the previous research conducted in Sarasota County, U.S., the elderly person living alone is extremely vulnerable to natural hazards [51]. Therefore, we followed this concept and took the solitary elderly population as a variable. The physically and mentally challenged population demands extra resources for their special needs [44,57]. Simultaneously, insufficient mobility will also augment the difficulty of evacuation and increase the probability of encountering the obstacle [58,59]. Age is one of the most effective and general variables for social vulnerability, similar to population density [5,60]. The children < 5 years old, elderly > 65 years old, and the aging index all have been widely applied in previous research [57,61,62]. These factors can represent the population proportion of lacking financial and information resources. Education level significantly connects to the ability of information understanding. It also has a direct effect on the potential resources for coping with natural hazards [21,44,48,52,53,61]. Thus, we adopted the population without a high school diploma as a variable.

The adaptive capacity component in SERV represents part of resilience, which is also aggregated by socioeconomic variables. We selected 12 most cited variables according to the previous studies and demonstrated in Table 2. The financial resource is an essential variable for adaptive capacity [63]. Income is a direct variable that can measure financial ability [64]. Therefore, our research took the annual income as the variable. According to previous research, adaptive capacity is significantly related to learning and acquiring information [10]. Several studies take the population with a college diploma as a variable [65,66]. As a result, we adopted the population with a college diploma as a variable. Moreover, previous research shows that the working population can attract political support and secure economic resources for recovery [63,65]. In other words, the working population has a positive influence on resilience and adaptive capacity. Subsequently, political engagement has a positive relationship with economic recovery [67]. Our study applied the voter to represent political engagement. Furthermore, community engagement represents the social bonds and the connections within the community. Community engagement also has a positive correlation to self-help when facing natural hazards [67]. The number of social-civic groups is significantly related to community engagement and relevant to adaptive capacity [44,68]. Moreover, studies regard the capacity of emergency shelters as an element of resilience and adaptive capacity [27,65,69,70]. Therefore, we adopted the capacity of emergency shelters as the variable. Numerous studies suggest that medical resources and capacity are essential for resilience and adaptive capacity [27,40,53,65,71,72]. Accordingly, we adopted four different variables. These variables are the number of healthcare facilities, number of licensed medical personnel, number of hospital beds, and number of pharmacies. Moreover, studies show that emergency services and ambulances are also crucial variables [29,65]. Undoubtedly, emergency response is a central element for adaptive capacity. Consequently, we adopted the number of emergency service stations and the number of ambulances as our variables.

The variables in this research have multicollinearity, which would create redundancy for our analysis. Therefore, we used PCA to aggregate the correlated variables. We used two kinds of statistical tests to examine the result of PCA. The first kind is the Kaiser-Meyer-Olkin (KMO) Test. The value of KMO can evaluate the correlations between variables. The acceptable threshold is 0.6. Subsequently, Bartlett’s test of sphericity can examine whether a few principal components can represent the redundancy of the dataset. The threshold of this test is whether the *p-*value less than 0.05 or not. After examining the result of PCA, there are two approaches to determine the number of principal components. One of the approaches is according to the starting point of the curve’s elbow in the scree plot. The other one is according to the eigenvalue. We applied the Varimax rotation with Kaiser normalization to extract the principal component, which eigenvalue is larger than 1.

### 2.3. Spatial Pattern Analysis and GWR

In order to explore the spatial difference, we must first detect the spatial pattern. Therefore, we apply two different scales of spatial pattern analysis approach. The global Moran’s *I* can detect the spatial pattern of vulnerability and resilience. According to the result of Moran’s *I*, the distribution pattern will be classified into random (Moran’s *I* ≅ 0), clustered (Moran’s *I* > 0), or dispersed (Moran’s *I* < 0). Equation (3) illustrates how the global Moran’s *I* was calculated [73,74].

(3) $$\mathrm{Moran}’s\;I\;=\;\frac{N}{\sum_{i=1}^{N}\sum_{j=1}^{N}W_{ij}}\times \frac{\sum_{i=1}^{N}\sum_{j=1}^{N}W_{ij}(X_{i}-\bar{X})\left(X_{j}-\bar{X}\right)}{\sum_{i=1}^{N}{\left(X_{i}-\bar{X}\right)}^{2}}$$

After detecting the distribution pattern through the global Moran’s *I*, we can visualize the pattern by a local indicator of spatial autocorrelation (LISA). The results of LISA can distinguish four different patterns. High-High (H-H) clustered means all the spatial units of a specific area have a high value. In contrast, the Low-Low (L-L) clustered represents all the units in a particular area that has a low value. The High-Low and Low-High outliers stand for the situation in which a high-value unit surround by low-value units and a low-value unit surround by high-value units, respectively. Equation (4) demonstrates how LISA was calculated [75].

(4) $$\mathrm{LISA}\;\frac{\left(X_{i}-\bar{X}\right)}{\sum_{i=1}^{N}{\left(X_{i}-\bar{X}\right)}^{2}}\sum_{j=1}^{N}W_{ij}\left(X_{j}-\bar{X}\right)$$

It is crucial to ensure the SERV can reflect the ground truth. Therefore, we used the Spatial Multivariable Regression and the authentic natural hazard events to validate the SERV. GWR is one of the modified models of spatial ordinary least squares (OLS). The original OLS regards the entire area as a single unit [76]. However, the spatial difference among the spatial units is unneglectable. The spatial difference will create a spatial nonstationary that will bias the result [77]. The GWR can solve the spatial nonstationary by creating several kernels with different bandwidths in the study area [54]. Equations (5) to (8) will illustrate how GWR was calculated [78,79].

(5) $$\mathrm{GWR}:y_{i}\left(u\right)={\hat{\beta }}_{0i}\left(u\right)+{\hat{\beta }}_{1i}\left(u\right)X_{1i}+{\hat{\beta }}_{2i}\left(u\right)X_{2i}+{\hat{\beta }}_{3i}\left(u\right)X_{3i}+\ldots +{\hat{\beta }}_{mi}\left(u\right)X_{mi}$$

(6) $${\hat{\beta }}_{mi}\left(u\right)={\left(X^{T}W\left(u\right)X\right)}^{-1}X^{T}W\left(u\right)y$$

(7) $$W\left(u\right)=\left[\begin{array}{ccc} w_{1}\left(u\right) & 0 & \begin{array}{cc} 0 & 0 \end{array}\\ 0 & w_{2}\left(u\right) & \begin{array}{cc} 0 & 0 \end{array}\\ 0 & 0 & \begin{array}{cc} \ddots & 0 \end{array}\\ 0 & 0 & \begin{array}{cc} 0 & w_{n}\left(u\right) \end{array} \end{array}\right]$$

(8) $$w_{n}\left(u\right)=e^{-0.5{\left[\frac{d_{n}\left(u\right)}{h}\right]}^{2}}$$

## 3. Results

### 3.1. Spatial Pattern of SERV

The SERV and its components (exposure, sensitivity, and adaptive capacity) are aggregated by several variables. In order to provide a more comprehensive explanation, we have to analyze the spatial pattern of all variables. We notice that most of the variables are correlated. Therefore, we apply the PCA to reduce the redundancy. The effectiveness test of the PCA is demonstrated in Table 3. The result of the PCA is acceptable according to the KMO and Bartlett’s test of sphericity. Both sensitivity and adaptive capacity are eventually aggregated into four principal components by PCA.

In the sensitivity part (Table 4), the principal component (a) is made up of the population without a high school diploma, aging index, standardized female population, solitary elderly population, physically and mentally challenged population, and elderly > 65 years old. The principal component (b) of the sensitivity contains the indigenous population ratio, MLI households, and children < 5 years old. The principal component (c) includes population density and dependency ratio. The principal component (d) of the sensitivity includes the foreign residents and laborers only.

Regarding the adaptive capacity part (Table 4), the principal component (e) composes of annual income, voter, number of social-civic groups, population with a college diploma, and working population. The principal component (f) of adaptive capacity includes the capacity of emergency shelters, the number of healthcare facilities, number of pharmacies. The principal component (g) of adaptive capacity consists of the number of licensed medical personnel and the number of hospital beds. The principal component (h) of adaptive capacity comprises the number of ambulances and the number of emergency service stations. The Moran’s *I* of most principal components are larger than 0. In addition, most of the *p*-value reach a statistically significant threshold. In other words, the pattern of most variables is a statistically significant cluster.

**Figure 3:**
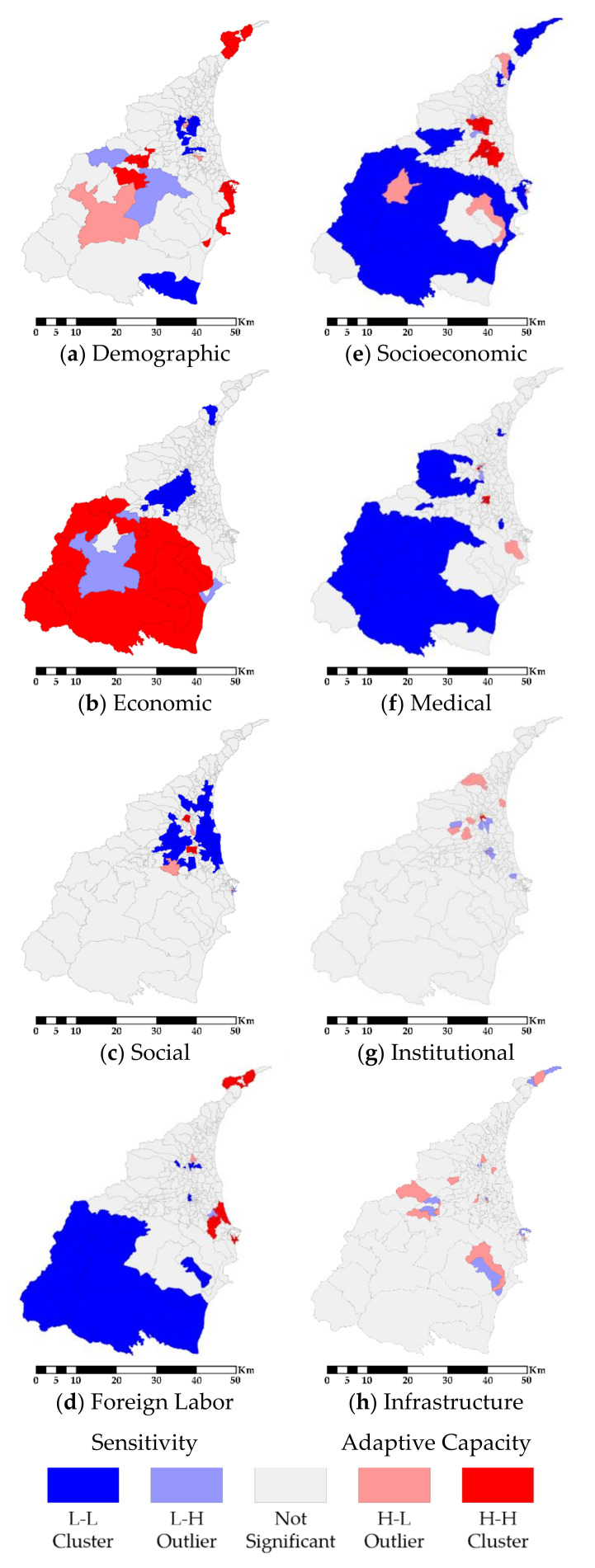
LISA results of all principal components.

Figure 3 illustrates the spatial cluster for each principal component. In the sensitivity part, L-L clusters are mostly located in the plain areas for components (a), (b), and (c), while they are located in the mountain areas for component (d). On the contrary, H-H clusters are mainly located in the coastal harbor areas for components (a) and (d), and they are also located in the mountain areas for component (b). This result reveals that the coastal harbor areas have higher sensitivity in demographic and foreign labor domains, but the mountain areas have relatively high sensitivity in the economic domain. The principal component (d) represents nursing, fishery, and maritime laborers. It shows L-L clusters in mountain areas and H-H clusters in coastal areas for component (d). One of the reasons is that most of the fishery and maritime laborers congregate in the surrounding areas of north Toucheng and south Su’ao Harbors. Nevertheless, foreign nursing laborers distribute dispersedly throughout the entire Yilan County. As a result, the foreign nursing labor creates no spatial cluster.

In the adaptive capacity part (Figure 3), we notice that the urban regions in the plain areas have H-H clusters for components (e) and (f), which represent the socioeconomic and medical domains, respectively. However, the mountain areas show the opposite result. The mountain areas have lower socioeconomic and medical domains of adaptive capacities. In addition, regarding the principal component (g) and (h), both Moran’s *I* and LISA show no significant spatial cluster. This means the distributions of the institutional and infrastructure domains are random.

**Figure 4:**
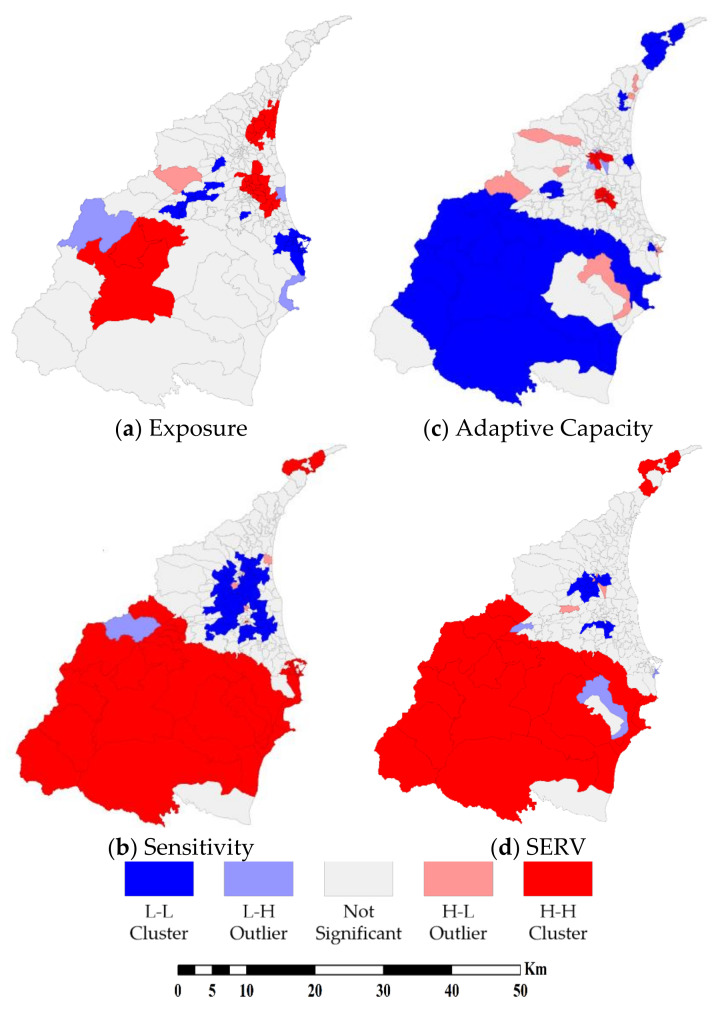
Results of LISA.

We aggregate principal components into sensitivity and adaptive capacity, respectively. Subsequently, we apply Moran’s *I* and LISA to detect the spatial pattern. Table 5 demonstrates the result of global spatial autocorrelation analysis. The results show that exposure, sensitivity, adaptive capacity, and SERV have significant clusters. Additionally, all *p-*values indicate the cluster is statistically significant. Then, we use LISA to visualize the location of clusters in Figure 4.

Figure 4a shows the spatial clusters of the exposure (physical vulnerability). There are two H-H clusters in the plain areas. The elevation is one of the main reasons. The elevation of the plain areas is lower in Yilan County. Moreover, a major river flows through the center of the plain areas. Most of the runoff will gather in the plain areas before entering the Pacific Ocean. This situation leads the plain areas to become higher exposed to flood hazards. For another area, the center of the mountain areas is also H-H cluster. Because of the high elevation and steep slope in the mountain areas, the intensive precipitation during the typhoon season often triggers serious soil erosion and sediment loads. Consequently, mountain areas have high exposure to debris flow hazards. Moreover, the rural regions and the part of the southern coastal areas are the L-L clusters of the exposure. The rural regions lay between the mountain areas and the plain areas. These areas have relatively mild elevation and gentle slopes. Therefore, both the potentials of flood and debris flow are lower.

Figure 4b,c demonstrate the clusters of sensitivity (social vulnerability) and adaptive capacity (resilience). For the sensitivity, most of the H-H clusters gather in mountain areas and coastal harbors. Most of the plain areas are the L-L clusters of sensitivity. As for the adaptive capacity, mountain areas gather most of the L-L clusters of adaptive capacity. In the center of the plain areas, urban regions show H-H clusters of the adaptive capacity. Subsequently, Figure 4d demonstrates the spatial distribution pattern of the SERV. The mountain areas are H-H clusters of SERV. In contrast, the urban regions in the plain areas are the L-L clusters of the SERV. The results of SERV reveal that there are higher exposure and sensitivity in mountain areas with lower adaptive capacity. In contrast, the adaptive capacity in urban regions is higher, but the exposure and sensitivity are relatively low. According to the result of LISA, it is obvious that there is a significant difference between mountain and plain areas in Yilan County.

### 3.2. GWR

**Figure 5:**
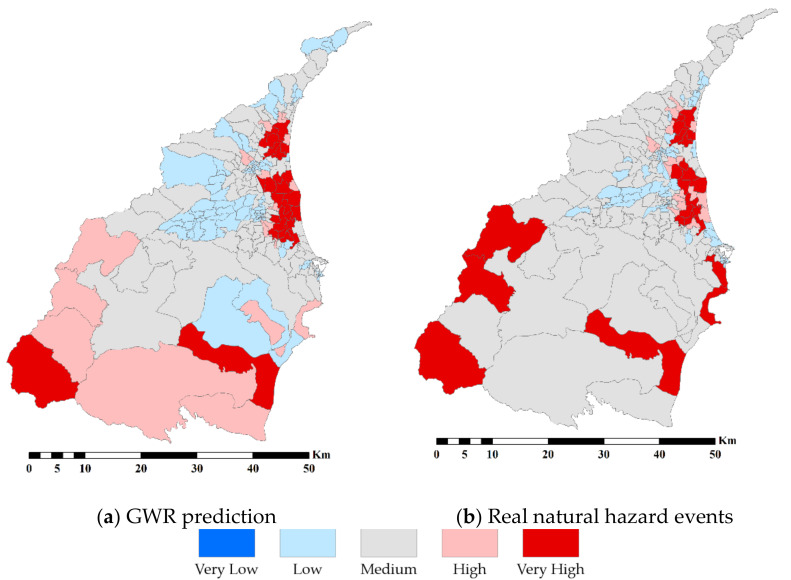
The standardized value of GWR prediction and real natural hazard events.

In order to understand the effectiveness of the SERV model, we apply the GWR to analyze this result. The authentic natural hazards, including flood and debris flow events extracted from the national database, are used to validate the SERV. Figure 5a shows the result of the standardized value of GWR prediction to natural hazards. We compare the spatial distributions of the predicted value in Figure 5a and real natural hazard events in Figure 5b to examine whether the SERV can reflect the ground truth. Both spatial distributions are mostly identical to each other. In addition, based on the result of GWR in Table 6, the R^2^ and adjusted R^2^ are 0.696 and 0.501, respectively. This result shows that the SERV we built is acceptable.

Although it seems the SERV has the acceptable explanatory ability, the evaluation we had mentioned above fails to prove the SERV is unbiased. The spatial pattern of the standardized residual is an indicator for evaluating the model bias. Therefore, we apply the global spatial autocorrelation to examine the distribution pattern of standardized residuals. According to Table 6, the Moran’s *I* of the standardized residuals is -0.055. Simultaneously, the *p-*value is 0.194, which does not reach a statistically significant threshold. The result of global spatial autocorrelation shows that the distribution pattern of the standardized residuals is random. In other words, the SERV we built is unbiased and has acceptable explanatory ability and effectiveness.

## 4. Discussion

Our research aims to explore the spatial relationship between vulnerability and resilience to natural hazards, including flood and debris flow events. In order to achieve the goal, we apply the SERV model. According to the R^2^ and adjusted R^2^, the SERV is valid. Through the spatial distribution of the standardized residual of GWR, the SERV is unbiased. Subsequently, the PCA generates four principal components for sensitivity and adaptive capacity, respectively. The results of PCA and LISA show that mountain areas are higher sensitivity and lower adaptive capacity. Mountain areas are H-H clusters of component (b) and L-L clusters of components (e) and (f). This spatial distribution indicates that mountain areas have relatively inferior development in the socioeconomic domain. The results of PCA and LISA indicate that the urban regions in plain areas are lower sensitivity and higher adaptive capacity. Urban regions in plain areas are L-L clusters of component (a), (b), and (c) and H-H clusters of components (e) and (f). These spatial patterns indicate that urban regions in plain areas have relatively favorable socioeconomic development. The results of PCA and LISA indicate that the situation in the urban regions is the opposite situation to mountain areas. A significant spatial difference exists between mountain and plain areas.

According to previous studies, the topography is one significant factor affecting the spatial distribution of vulnerability and resilience to natural hazards [4,27,37,66]. Regarding the vulnerability, the previous study indicates that the mountain areas have higher sensitivity than plain areas [4]. The plain areas have better socioeconomic development than mountain areas [4]. The mountain areas have inferior socioeconomic development [37]. Regarding the adaptive capacity, the previous research has also shown a similar result [8]. The majority of states in the mountains have the lowest resilience and adaptive capacity [8,66]. Indeed, situations of the different countries might not be similar to each other, and the development level might have significant differences from place to place and from time to time. Thus, it shows that the topography has influenced the spatial pattern.

The distribution patterns of the component (e) and (f) are remarkable. The component (e) and (f) in our research represent the socioeconomic and medical domains of adaptive capacity. These distribution patterns should follow the concept of “equally distribute”, which allows the civilian to have an equal chance to access these resources. Nevertheless, the spatial distributions of components (e) and (f) fail to focus on the mountain areas where most needed. Consequently, these resources cannot equally support these areas with inferior socioeconomic development. This means those vulnerable areas do not obtain enough resources to mitigate their disadvantages. In other words, the distribution of socioeconomic and medical aspects of adaptive capacity somehow jeopardizes the mountain areas instead of helping.

In summary, we discover that mountain areas have a higher sensitivity and lower adaptive capacity. For another part of our case study area, plain areas are the opposite situation. The plain areas have a lower sensitivity and higher adaptive capacity. The unequal distributions of socioeconomic advantages and healthcare resources increase the vulnerability of the mountain areas.

## 5. Conclusions

Compared to other administrative areas in eastern Taiwan, our case study area has more opportunities for socioeconomic development because it is closely adjacent to Taipei City. Nevertheless, the development of Yilan County is significantly uneven. The result of the spatial autocorrelation analysis shows a significant difference between the plain and mountain areas. The plain areas have better socioeconomic development than the mountain areas. One of the most noticeable spatial differences is the distinct topography conditions. The topography plays an essential role in the distribution of exposure, sensitivity, and adaptive capacity. Most of the traffic nodes gather only in plain areas. The mountain areas have low connectivity to main traffic nodes. The topography becomes an obstacle for people to travel into mountain areas. Another drawback created by the topography is the higher cost for industrial development. The steep slopes in the mountain areas will undoubtedly augment the difficulty and cost. All these disadvantages make the mountain areas into higher sensitivity and lower adaptive capacity. Moreover, the mountain areas also have high exposure to the potential of debris flows. Accordingly, mountain areas are extremely vulnerable. In contrast, plain areas have relatively favorable conditions for living and socioeconomic development. The advantage leads the plain areas into a lower vulnerability and higher adaptive capacity. Despite the fact that parts of the plain areas have higher exposure to the potential of floods, their higher adaptive capacity can compensate for this disadvantage. Therefore, situations between mountain and plain areas are significantly different. Although there is a significant spatial difference in our case study area, some previous studies indicate a different result. It shows that not all the urban regions have a higher level of adaptive capacity, and not all rural regions have a lower level of adaptive capacity. The domains of adaptive capacity are various in different places. Urban regions dominate the economy; however, the rural regions still have other advantages such as social bonds and community engagement. According to previous studies and our research, topography is an important factor. The topography highly affects the spatial distribution of vulnerability and resilience to natural hazards. The result shows that the situation varies from place to place.

## Figures and Tables

**Table 1:** Indicators and variables of sensitivity.

Indicators and Variables of Sensitivity	Moran’s *I*	*p*-Value
Population Density	0.634	<0.05
Standardized Female Population	0.582	<0.05
Middle/Low-income (MLI) Household	0.488	<0.05
Dependency Ratio	0.420	<0.05
Foreign Residents and Laborers	0.244	<0.05
Indigenous Population Ratio	0.650	<0.05
Solitary Elderly Population	0.197	<0.05
Physically and Mentally Challenged Population	0.198	<0.05
Children < 5 Years Old	0.433	<0.05
Elderly > 65 Years Old	0.337	<0.05
Aging Index	0.107	<0.05
Population without High School Diploma	0.706	<0.05

**Table 2:** Indicators and variables of adaptive capacity.

Indicators and Variables of Adaptive Capacity	Moran’s *I*	*p*-Value
Annual Income	0.365	<0.05
Population with a College Diploma	0.556	<0.05
Working Population	0.277	<0.05
Voter	0.388	<0.05
Number of Social-Civic Groups	0.124	<0.05
Capacity of Emergency Shelters	0.063	0.10
Number of Healthcare Facilities	0.407	<0.05
Number of Licensed Medical Personnel	0.017	0.41
Number of Hospital Beds	−0.013	0.81
Number of Pharmacies	0.283	<0.05
Number of Emergency Services Stations	−0.068	0.12
Number of Ambulances	−0.018	0.69

**Table 3:** Effectiveness test of PCA.

Component	KMO	*p*-Value of Bartlett’s Test of Sphericity	Total Variables Explained
Sensitivity	0.647	<0.05	77%
Adaptive capacity	0.622	<0.05	65%

**Table 4:** Spatial autocorrelation result of the principal components.

Sensitivity			
Component	Moran’s *I*	*p*-Value	Domain
Principal component (a)	0.335	<0.05	Demographic
Principal component (b)	0.584	<0.05	Economic
Principal component (c)	0.594	<0.05	Social
Principal component (d)	0.224	<0.05	Foreign Labor
**Adaptive capacity**			
**Component**	**Moran’s *I***	***p*-Value**	**Domain**
Principal component (e)	0.563	<0.05	Socioeconomic
Principal component (f)	0.286	<0.05	Medical
Principal component (g)	0.002	0.83	Institutional
Principal component (h)	−0.052	0.18	Infrastructure

**Table 5:** Spatial autocorrelation result of the exposure, sensitivity, adaptive capacity, and SERV.

Component	Moran’s *I*	*p*-Value
Exposure (E)	0.477	<0.05
Sensitivity (S)	0.584	<0.05
Adaptive Capacity (AC)	0.406	<0.05
SERV ([E+S]-AC)	0.414	<0.05

**Table 6:** Summary of GWR.

Neighbors	R^2^	Adjusted R^2^	Moran’s *I* of StdResid	The *p*-Value of Moran’s *I*
31	0.696	0.501	−0.055	0.194

## Data Availability

The data presented in this study are available on request from the corresponding author.
